# Correlation of Apical Fluid-Regulating Channel Proteins with Lung Function in Human COPD Lungs

**DOI:** 10.1371/journal.pone.0109725

**Published:** 2014-10-17

**Authors:** Runzhen Zhao, Xinrong Liang, Meimi Zhao, Shan-Lu Liu, Yao Huang, Steven Idell, Xiumin Li, Hong-Long Ji

**Affiliations:** 1 Department of Cellular and Molecular Biology, University of Texas Health Science Center at Tyler, Tyler, Texas, United States of America; 2 Medicine, University of Texas Health Science Center at Tyler, Tyler, Texas, United States of America; 3 Texas Lung Injury Institute, University of Texas Health Science Center at Tyler, Tyler, Texas, United States of America; 4 Department of Molecular Microbiology and Immunology, Bond Life Sciences Center, University of Missouri, Columbia, Missouri, United States of America; 5 Department of Obstetrics and Gynecology, St. Joseph's Hospital and Medical Center, Phoenix, Arizona, United States of America; 6 Xinxiang Medical University, Xinxiang, Henan, China; The Ohio State University, United States of America

## Abstract

Links between epithelial ion channels and chronic obstructive pulmonary diseases (COPD) are emerging through animal model and in vitro studies. However, clinical correlations between fluid-regulating channel proteins and lung function in COPD remain to be elucidated. To quantitatively measure epithelial sodium channels (ENaC), cystic fibrosis transmembrane conductance regulator (CFTR), and aquaporin 5 (AQP5) proteins in human COPD lungs and to analyze the correlation with declining lung function, quantitative western blots were used. Spearman tests were performed to identify correlations between channel proteins and lung function. The expression of α and β ENaC subunits was augmented and inversely associated with lung function. In contrast, both total and alveolar type I (ATI) and II (ATII)-specific CFTR proteins were reduced. The expression level of CFTR proteins was associated with FEV1 positively. Abundance of AQP5 proteins and extracellular superoxide dismutase (SOD3) was decreased and correlated with spirometry test results and gas exchange positively. Furthermore, these channel proteins were significantly associated with severity of disease. Our study demonstrates that expression of ENaC, AQP5, and CFTR proteins in human COPD lungs is quantitatively associated with lung function and severity of COPD. These apically located fluid-regulating channels may thereby serve as biomarkers and potent druggable targets of COPD.

## Introduction

Mucus hypersecretive COPD is the fourth and will be the third leading cause of death by 2020 worldwide [1]–[3]. As a widely acknowledged heterogeneous disease, COPD encompasses small airway disease, emphysema, and chronic obstructive bronchitis. Notwithstanding the complexity, genetic studies have associated several genes with COPD, including SOD3, GSTM1, TGFB1, TNF, GSTP1, etc [4]–[6].

Defective mucin hydration has been confirmed in the airways of cystic fibrosis sufferers. Abnormal bioelectric properties including hyperactive ENaC activity and deficient cystic fibrosis transmembrane conductance regulator (CFTR) are well-known in cystic fibrosis lungs [7], [8]. CFTR interactively regulates other proteins' activities [9]–[11]. These epithelial channel proteins, together with aquaporins and Na^+^-K^+^-ATPases finely adjust luminal surface fluid in the airways and air spaces [12], [13].

Mall and co-workers successfully established a “COPD-like” mouse strain by genetically over-expressing β ENaC in airway epithelia [14]–[17]. These transgenic mice exhibit dehydrated airways and severe mucus obstruction in the trachea. In addition, the authors noted goblet cell hyperplasia and neutrophilic inflammation, commonly observed in COPD [14], [18], [19]. In surviving mice, increased mucus concentration and delayed mucus transport in the conducting airways were observed [14]. Clinical pathologic examination revealed that chronic mucus obstruction was found in human distal airways and lungs, accompanied by goblet cell metaplasia, increased mucin expression, persistent neutrophilic airway inflammation, and transient eosinophilic airway filtration [20]. Furthermore, mice over-expressing multiple ENaC subunits developed emphysema with increased lung volumes, distal airspace enlargement, and decreased lung compliance [14], [18]. The correlations between ENaCs and lung function in human COPD, however, are not yet known. Given the inter-regulation of ENaC, CFTR, and aquaporins, we aimed to quantitatively analyze the expression of fluid-regulating ion channel proteins, including ENaC, CFTR, and AQP5 in human COPD lungs. We then sought to correlate their expression levels with declined lung function. Our novel results demonstrate that the expression of these channel proteins in human COPD lungs is significantly associated with declined spirometry test, gas exchange, and severity of COPD.

## Materials and Methods

### Human lung tissues and clinical data

The use of lung tissues curated by the Lung Tissue Research Consortium (LTRC), National Institute of Health has been approved by the Institutional Review Board (EXEMPT 07–013) of the University of Texas Health Science Center at Tyler. Written informed consents from the donor or the next of kin were obtained for use of these samples in research. Frozen human lung tissue blocks for protein extractions, fixed human lung slices for histology, and anonymous lung function data from COPD patients and healthy individuals were provided by the LTRC. Of the thirty four smokers, twenty-four were diagnosed with moderate to severe COPD/emphysema, and ten healthy subjects were controls according to FEV1 pre-bronchodilator % predicted values (FEV1pd1a): control ≥80%, moderate 50–80%, and severe <50%. The patients' demographics, spirometric results, and gas diffusion capacity are summarized in Table 1.

**Table 1:** Patient demographics.

	*Control*	*Moderate*	*Severe*	*P value*
*Sex, M/F*	*5/5*	*9/3*	*7/5*	*0.474*
*Age, yr*	*65.5 (60.3, 77.5)*	*61.5 (57.5, 72)*	*51 (46.8, 62) ^a,b^*	*0.01*
*Height, in*	*66.5 (62, 72)*	*68.5 (66.3, 70)*	*66.5 (64.3, 69)*	*0.631*
*Weight, lb*	*175 (145, 200)*	*195 (155, 220)*	*155 (125, 180)*	*0.162*
*Cigarette, pack-yrs*	*29 (18.8, 55)*	*45 (33, 59)*	*41 (32, 81.5)*	*0.380*
*SF12 score, physical*	*37.1 (30.5, 56.4)*	*30.7 (25.3, 39.3)*	*26.8 (24.9, 30.3) ^a^*	*0.01*
*St. George's score*	*20.2 (4.5, 49.6)*	*47.2 (31.4, 67.6)*	*53.5 (49.9, 63) ^a^*	*0.015*
*% predicted, FEV1 pre*	*93.5 (85.5, 97.5)*	*61.5 (56.5, 69.5) ^a^*	*20 (17.5, 23.5) ^a,b^*	*<0.0001*
*% predicted, FEV6 pre*	*101 (90.3, 115.3)*	*79.5 (66.3, 89) ^a^*	*38.5 (34.3, 48.8) ^a,b^*	*<0.0001*
*% predicted, FVC pre*	*100 (89.5, 110.8)*	*82 (64.3, 91) ^a^*	*51 (42.3, 62) ^a,b^*	*<0.0001*
*% predicted, PEFR pre*	*98.5 (89.5, 112.3)*	*76.5 (67.3, 81.8)*	*30.5 (26.3, 32.8) ^a,b^*	*<0.0001*
*Pre-FEV1/FVC ratio*	*0.7 (0.68, 0.8)*	*0.6 (0.5, 0.7) ^a^*	*0.3 (0.23, 0.38) ^a,b^*	*<0.0001*
*% predicted, FEV1 post*	*97.5 (89, 106)*	*67 (65, 76) ^a^*	*19.5 (16.5, 22.5) ^a,b^*	*<0.0001*
*% predicted, FEV6 post*	*108.5 (103, 120)*	*88 (80, 90) ^a^*	*42 (39, 47) ^a,b^*	*0.002*
*% predicted, FVC post*	*107.5 (102, 117)*	*91 (87, 96) ^a^*	*50 (38, 62) ^a,b^*	*0.002*
*% predicted, PEFR post*	*108 (94.5, 124.8)*	*88 (69, 90) ^a^*	*28 (22.3, 48.8) ^a,b^*	*<0.0001*
*Post-FEV1/FVC ratio*	*0.7 (0.6, 0.73)*	*0.6 (0.5, 0.6) ^a^*	*0.35 (0.23, 0.4) ^a,b^*	*0.003*
*Perdlco*	*87 (64.3, 111.5)*	*58 (31, 75)*	*45 (25, 55.5) ^a^*	*0.012*

Values are medians (25^th^, 75^th^ percentile). Perdlco, severity classification of DLCO abnormality (continuous). Severity was grouped using Gold standard according to REV1pd1a (see Methods): Controls ≥80%; Moderate ≥50 and <80%; Severe <50%. Statistical analysis was performed using Mann-Whitney U test and Kruskal-Wallis ANOVA. P value <0.05 was considered significant. ^a^P<0.05 compared with Control group; ^b^P<0.05 versus moderate stage.

### Questionnaires

All subjects completed standardized questionnaires to assess respiratory history and symptoms, respiratory medicines, smoking history, family history and other medical histories. The LTRC database curated by four clinical centers (The Mayo Clinic chaired by Dr. Andrew H. Limper, Temple University by Dr. Gerard J. Criner, University of Michigan by Dr. Fernando J. Martinez, and University of Pittsburgh by Dr. Frank C. Sciurba) includes data from CT scans, chest X-rays, tissue types, and both final clinical and pathological diagnoses.

### Spirometry

Spirometry was performed in a standardized manner. The complete study forms for this LTRC project can be obtained from the LTRC website (http://www.ltrcpublic.com/). The summarized spirometric results are included in Table 1. In addition, a list of final clinical diagnoses and final pathological diagnoses can be found on the website.

### Western blots

Human lung protein extracts were prepared as follows. Frozen lung tissues were chopped into small pieces with fine scissors, homogenized in RIPA buffer supplemented with protease inhibitor (Roche) and incubated for 2 h at 4°C with agitation. The homogenates were centrifuged at 16,000×g for 15 min at 4°C. Protein concentrations were determined by the BCA assay and analyzed by Western blotting (100 µg per lane). 6%, 10%, and 15% SDS-PAGE gels were used for CFTR, AQP5, and Pro-SPC, respectively, while 8% SDS-PAGE gel was used for all other proteins. Proteins were electrotransferred to PVDF membranes (Bio-RAD, Hercules CA). The membranes were blocked with 5% non-fat dry milk in TBST buffer, and then incubated overnight at 4°C with the following specific primary antibodies: anti-human α ENaC (Santa Cruz, sc-22239, 1∶250), anti-human β ENaC (Santa Cruz, sc-21013, 1∶200), anti-human γ ENaC (Abcam, ab3468, 1∶1400), anti-human SOD3 (Santa Cruz, sc-32219, 1∶700), anti-human AQP5 (Santa Cruz, sc-28628, 1∶500), anti-pro-SPC (Millipore, AB3786, 1∶800), and anti-human CFTR antibodies (R & D Systems, MAB25031, 1∶400). HRP-conjugated secondary antibodies were added post-washing with TBST buffer. Signals were detected using ECL reagents (Millipore, WBKLS0500). ENaC and CFTR proteins are expressed in both human ATI and ATII cells and contribute to ion transport [21]–[24]. To quantitatively compute expression levels relative to β actin (total proteins), AQP5 (proteins in ATI cells), and pro-SPC (proteins in ATII cells), the membranes were stripped and re-probed with corresponding antibodies. Densitometry was analyzed with QUANTITY ONE (Bio-Rad).

### Statistical analysis

Clinical data are presented as median values (25^th^, 75^th^ percentile). Mann-Whitney U test and Kruskal-Willis ANOVA computations were utilized to analyze the differences in the mean values using Origin Pro 8.5. Real-time RT-PCR and Western blot data were analyzed using one-way ANOVA. P<0.05 was considered statistically significant. To examine the correlation between paired clinical and laboratory data, we computed the Spearman's product moment correlation coefficient values. The correlation was considered significant when P<0.01 for the Spearman coefficient and P<0.05 for correlation strength.

## Results

### Expression of SOD3 and alveolar epithelial biomarkers

**Figure 1:**
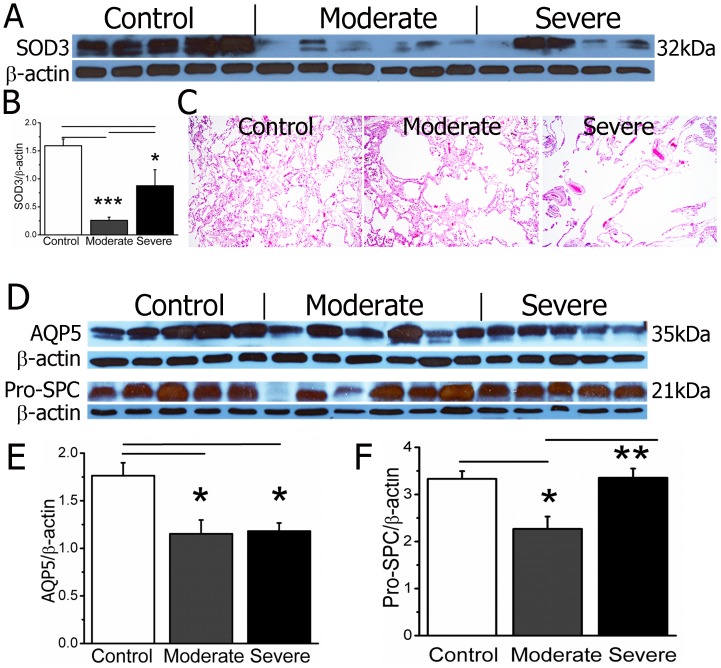
SOD3, AQP5, and pro-SPC proteins in control, moderate, and severe COPD lungs. **A**. Two bands (32 and 30 kDa) are recognized by a specific polyclonal antibody against human SOD3 (Santa Cruz, sc-32219) via Western blot. The blots were stripped and reprobed with a monoclonal anti-β actin antibody. **B**. Quantitative densitometry of SOD3 relative to corresponding β actin. One-way ANOVA. *P<0.05 and ***P<0.001 vs control or moderate group. N = 17. **C**. Representative images of control, moderate, and severe groups. H & E stain. 10×. **D**. AQP5 proteins are detected by a specific antibody against human AQP5 (Santa Cruz, sc-28628) via Western blot. Pro-SPC is detected (21 kDa) using a specific antibody (Millipore, AB3786). **E**. Densitometric measurements of AQP5 over β actin. N = 17. *P<0.05 versus controls. **F**. Relative pro-SPC protein expression. One-way ANOVA. *P<0.05 versus controls and **P<0.01 versus moderate group.

Extracellular superoxide dismutase (SOD3) is a major superoxide scavenger in the respiratory system. Antioxidants may protect the airways and lungs against excessive superoxide free radicals from tobacco and inflammatory cells. SOD3 proteins are reduced in COPD lung tissues [25], most likely from attenuating oxidative fragmentation of the extracellular matrix (i.g., collagen I, hyaluronan, and heparin sulfate) to protect against alveolar enlargement [26], [27]. SOD3 was associated with FEV1 in COPD patients [28]. This was supported by the association of inherited SOD3 mutations and FVC% in two population studies [29]. To evaluate the reliability of our samples, we set out to analyze SOD3 expression in lung tissues. Two bands (32 and 31 kDa) were recognized by a SOD3-specific antibody (Figure 1A). SOD3 proteins showed a significant decrease in both moderate and severe COPD lungs (Figure 1A & B). These observations were consistent with a recent report in the LTRC-provided samples [25], indicating that the tissues were suitable for protein assays. We therefore used SOD3 as a control in subsequent experiments and statistical analyses. SOD3 proteins were reduced to a lesser extent in moderate and severe COPD lung tissues compared with controls. The inflammatory responses may affect the translation and posttranslational modifications of SOD3 and ion channel proteins. Indeed, pulmonary inflammation is more severe in moderate versus severe COPD lungs, as determined by leukocyte infiltration, airspace flooding, and hemorrhage (Figure 1C).

To quantitate cell-specific expression of ENaC and CFTR, the translational levels of specific biomarkers for ATI (AQP5) and ATII (pro-SPC) epithelial cells were analyzed (Figure 1D). AQP5 exhibits a 30% reduction in COPD lungs (Figure 1E). However, reduction in pro-SPC to the same degree was only seen in moderate COPD lungs (Figure 1F). Therefore, to analyze the expression of ENaC and CFTR in ATI and ATII sub-populations, both absolute and compensated protein levels were computed (data follow).

### Augmented expression of ENaC proteins in human COPD lungs

**Figure 2:**
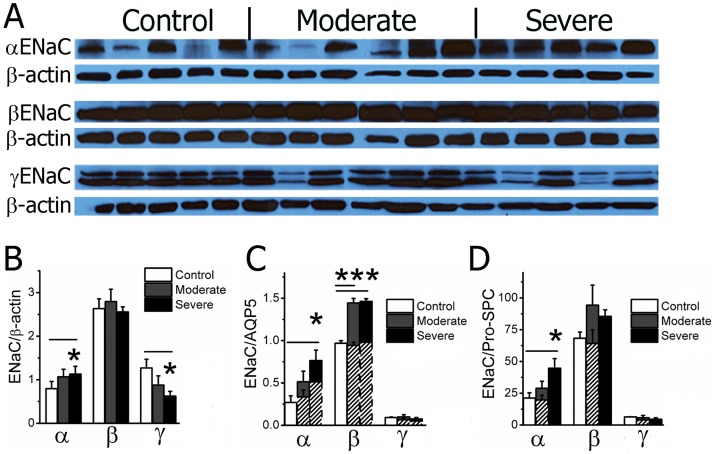
Expression of ENaC proteins. **A**. Representative Western blots. α (90 kDa), β (95 kDa) and γ ENaC protein signals are found in blots probed with specific antibodies against α (Santa Cruz, sc-22239), β (Santa Cruz, sc-21013), and γ (Abcam, ab3468) subunits. **B**. Total expression levels of ENaC subunits. β actin is used as a loading control. **C & D**. Expression of ENaC proteins in ATI and ATII cells. The expression levels of ENaC proteins related to AQP5 (**C**) and pro-SPC (**D**) were adjusted to compensate for the alterations in AQP5 and pro-SPC (dotted bars). One-way ANOVA. *P<0.05 and ***P<0.001 versus control groups.

Transgenic mice over-expressing ENaC had COPD-like phenotype [14]. We hypothesized that ENaC translation in COPD lungs is increased. Expression levels of the four ENaC subunits are not identical and are non-coordinately regulated by hormones [30]. The well-characterized anti-α and anti-β ENaC antibodies recognized a 90 kDa and a 95 kDa single band, respectively, by Western blot. By comparison, a specific anti-γ ENaC antibody identified two forms of proteins: full-length parental (95 kDa) and proteolytically cleaved protein (85 kDa) (Figure 2A). Average α ENaC level relative to corresponding β actin was significantly elevated, while γ ENaC level was reduced, in severe COPD lungs as determined by densitometry, (Figure 2B–D). ENaC proteins are expressed in both alveolar type I (ATI) and II (ATII) cells. Squamous ATI cells cover approximately 93% of airspace surface area (140 m^2^), while ATI cells account for up to 35% of the cell population [31]. In COPD/emphysema lungs, the airspace surface area was reduced by up to 75% due to alveolar enlargement [32], [33]. To examine whether the changes in ENaC expression in subpopulations of alveolar cells varied, we analyzed ENaC expression in ATI and ATII cells separately. Our data showed an increase in α ENaC protein in both ATI and ATII cells (Figure 2C & D). Expression of β ENaC was enhanced significantly in COPD lungs too (Figure 2C). In contrast, neither full-length nor trimmed peptide γ ENaC proteins underwent a significant change in both cell types (Figure 2C & D). Our results for the first time suggest that the expression of α and β ENaC proteins is augmented in human COPD lung tissues.

### Decreased CFTR proteins in COPD lungs

**Figure 3:**
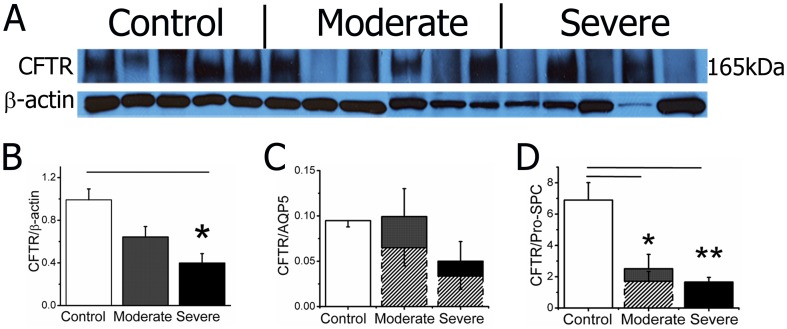
CFTR expression in COPD lungs. **A**. Western blots of CFTR expression. A predominant band at 165 kDa is detected by a specific anti-CFTR antibody (R&D Systems, MAB25031) in human lungs. β actin serves as a loading control. **B–D**. Densitometry of total CFTR expression (**B**), relative expression in ATI (**C**), and ATII (**D**) cells. Dotted bars show the values due to reduced expression of AQP5 and pro-SPC. One-way ANOVA. *P<0.05 and **P<0.01 versus control groups.

CFTR proteins are detected in human ciliated airway epithelial cells and both type I and II alveolar cells [21]. CFTR has been implicated in the early development of chronic bronchitis and emphysema [34]. Reduction in CFTR activity in vivo was found in COPD patients [35]. Furthermore, a recent publication reported an alteration in CFTR in COPD lungs [35]. To examine a potential association between CFTR proteins and decline in lung function, we quantitated CFTR expression at the translational level. As shown in Figure 3A, CFTR protein expression is positively correlated with COPD severity. We then sought to analyze CFTR expression in ATI and ATII cells. A significant reduction in CFTR signal in both cell types was observed (Figure 3C & D). Considering the alterations in the cellular biomarkers used for quantification, the CFTR expression level is likely overestimated. We compensated by adjusting the densitometric values of CFTR as shown with dotted bars (Figure 3C & D). The new computation suggests that the reduction of CFTR in ATII cells may predominately contribute to the observed decrease in total proteins.

### Total expression of SOD3 and ENaC proteins correlates with decline in lung function

**Figure 4:**
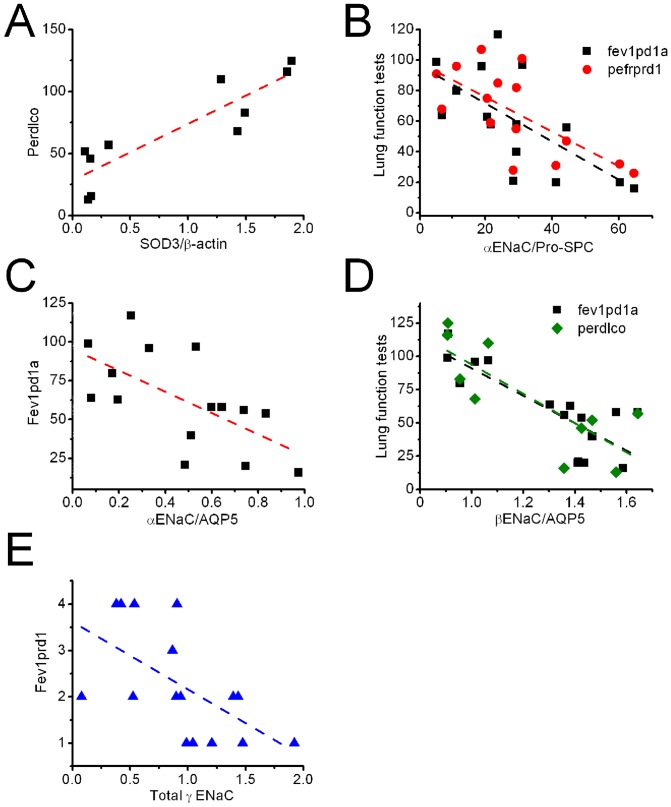
Association of spirometry and expression of SOD3 and ENaC in COPD lungs. **A**. Total SOD3 and perdlco. **B**. α ENaC in ATII cells and spirometry. **C**. α ENaC expressed in ATI cells and fev1pd1a. **D**. β ENaC in ATI cells and lung function test. **E**. Total γ ENaC level and fev1prd1.

Given the protective effects of SOD3 against COPD pathogenesis, and the association of SOD3 with FEV1 [26], [28], association between total SOD3 expression and lung function test was analyzed (Figure 4A). SOD3 expression level was related to perdlco, carbon monoxide diffusion capacity. Decreased value of perdlco in COPD may be due to large residual air stuck in the lungs and limited effective alveolar area.

ENaC is a major pathway for vectorial salt transport across the respiratory epithelium. Genetic and acquired modifications of ENaC were associated with fluid homeostasis at the luminal surface throughout the respiratory system (see review [36]). We asked whether there is a correlation between ENaC expression in lung tissues and clinical lung function test. Our results connected the amount of ENaC proteins with both spirometry and carbon monoxide diffusion capacity (Table 2). A stronger relationship between expression of ENaC subunits and decline in lung function was seen in ATI cells (correlation strength ranging from −68.5 to −109.8) compared with ATII pneumocytes (correlation strength ranging from −1.1 to −1.5), as reflected by the correlation strength (Table 2). Inverse associations between α ENaC in alveolar cells and two spirometric parameters, termed fev1pd1a and pefrprd1, were observed (Figure 4B & C). In addition to spirometry, β ENaC was associated with gas exchange across the gas-blood barrier (Figure 4D). Similarly, a negative correlation between γ ENaC and FEV1 was found (Figure 4E). Taken together, ENaC proteins (α, β, and γ) are negatively correlated with spirometry tests and diffusion capacity of carbon monoxide. In other words, decline in lung function is accompanied by a gradual increase in ENaC expression.

**Table 2:** Correlation of channel proteins with lung function.

	Spearman coefficient	P value	Correlation strength	P value
***ENaC***				
* Protein/β actin (* ***total*** *)*				
* γ ENaC vs fev1prd1*	*−0.690*	*0.003*	*−1.5*	*0.016*
* Protein/Pro-SPC (* ***ATII*** *)*				
* α ENaC vs fev1pd1a*	*−0.705*	*0.003*	*−1.3*	*0.007*
* vs pefrprd1*	*−0.833*	*0.01*	*−1.1*	*0.004*
* Protein/AQP5 (* ***ATI*** *)*				
* α ENaC vs fev1pd1a*	*−0.757*	*<0.0001*	***−68.5***	*0.003*
* β ENaC vs fev1pd1a*	*−0.773*	*<0.0001*	***−102.8***	*<0.0001*
* vs perdlco*	*−0.782*	*0.008*	***−109.8***	*0.005*
***CFTR***				
* Protein/β actin (* ***total*** *)*				
* vs fev1prd2*	*0.881*	*0.004*	***75.5***	*0.005*
* Protein/Pro-SPC (* ***ATII*** *)*				
* vs sf12pcs*	*0.867*	*0.003*	*2.9*	*0.034*
* vs fev1pd1a*	*0.778*	*0.003*	*9.5*	*<0.001*
* vs fev6prd1*	*0.9*	*<0.0001*	*7.7*	*0.012*
* vs fvcprd1*	*0.902*	*<0.0001*	*8.1*	*0.002*
* vs fev1prd2*	*0.929*	*0.003*	*10.6*	*0.006*
* vs fvcprd2*	*0.929*	*0.003*	*7.2*	*0.027*
* Protein/AQP5 (* ***ATI*** *)*				
* vs fev1prd2*	*0.929*	*<0.0001*	***927***	*0.001*
* vs fev6prd2*	*0.893*	*0.007*	***772***	*0.006*
* vs fvcprd2*	*0.952*	*<0.0001*	***722***	*<0.001*
***AQP5 (ATI)***				
* Protein/β actin (* ***total*** *)*				
* vs fev1pd1a*	*0.669*	*0.005*	***47.9***	*0.01*
* vs perdlco*	*0.903*	*<0.0001*	***72.2***	*<0.001*
***SOD3***				
* Protein/β actin (* ***total*** *)*				
* vs perdlco*	*0.879*	*<0.0001*	***45.7***	*<0.001*

Spearman correlation coefficient constants and P values were computed. Corresponding correlation strength was computed by fitting data with a linear regression function. fev1pd1a, FEV1 pre-bronchodilator % predicted (continuous); pefrprd1, PEFR post-bronchodilator % predicted; perdlco, severity classification of DLCO abnormality (continuous); fev1prd2, FEV1 post-bronchodilator % predicted (continuous); sf12pcs, SF12 physical component score; fev6prd1, FEV6 pre-bronchodilator % predicted; fev1prd2, FEV1 post-bronchodilator % predicted (continuous); fvcprd2, FVC post-bronchodilator % predicted. Additional expanded abbreviations can be found in Table 1. Spearman coefficients (between −1 and +1) were calculated using Origin Pro 8.5.

### Expression of water channels is proportional to spirometry

**Figure 5:**
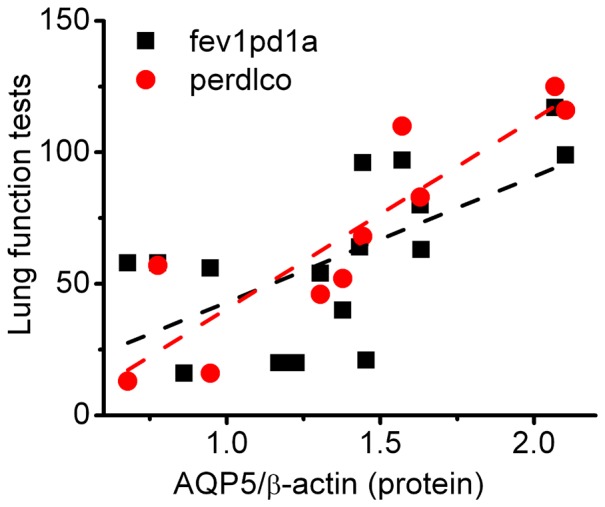
Correlation of aquaporin 5 with spirometry test. Dotted lines were created by linear regression analysis of the paired experimental data.

AQP5 is predominantly expressed in ATI cells, and to a lesser extent in airway epithelial cells and submucosal gland acinar cells [37]. AQP5 in the airways was associated with mucus overproduction and lower lung function [38]. Our analysis showed that AQP5 was related to carbon monoxide diffusion in addition to FEV1 (Figure 5). In contrast, we did not see the correlation between pro-SPC and lung function tests with this sample size.

### Positive correlation between lung function and CFTR abundance

**Figure 6:**
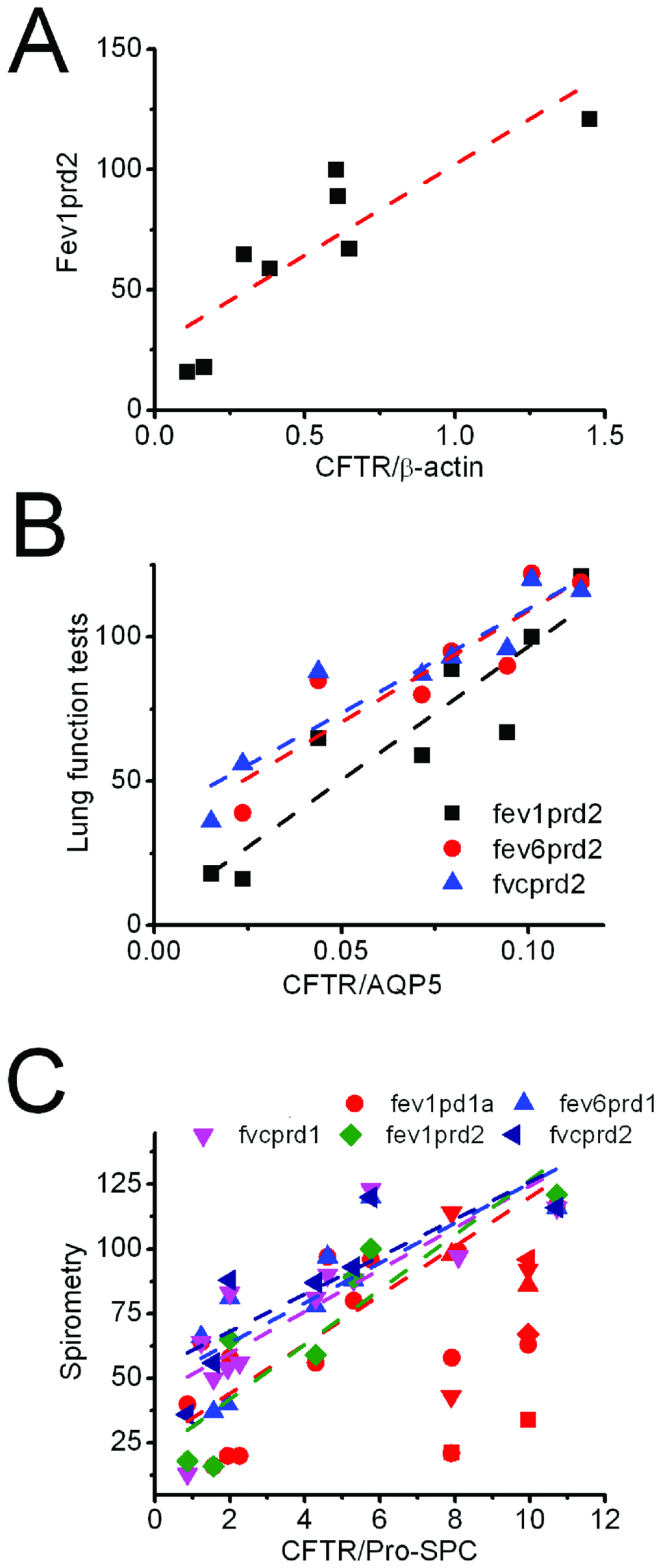
Positive correlation of CFTR with spirometry test in COPD lungs. **A**. Total CFTR expression and fev1prd2. **B**. Expression of CFTR in ATI cells and three spirometric parameters. **C**. CFTR proteins in ATII cells and spirometry test.

Delineation of the role of CFTR in COPD is ongoing [34]. To find the specific spirometry parameters associated with CFTR translation, we analyzed their correlation and found lung total CFTR expression was positively associated with fev1prd2 (Figure 6A). We further computed the Spearman correlation coefficient constant between lung function and the expression level of CFTR protein in two types of alveolar cells (Figures 5B & C). CFTR in ATI cells is positively correlated with FEV1, FEV6, and FVC measurements. In addition to these spirometry tests, CFTR in ATII cells correlated with physical and mental surveys (SF-12), and the correlation strength in ATI cells (from 722 to 927) was significantly greater than those in type 2 pneumocytes (from 2.9 to 10.6).

### COPD severity is associated with expression of SOD3, ENaC, and CFTR proteins

**Figure 7:**
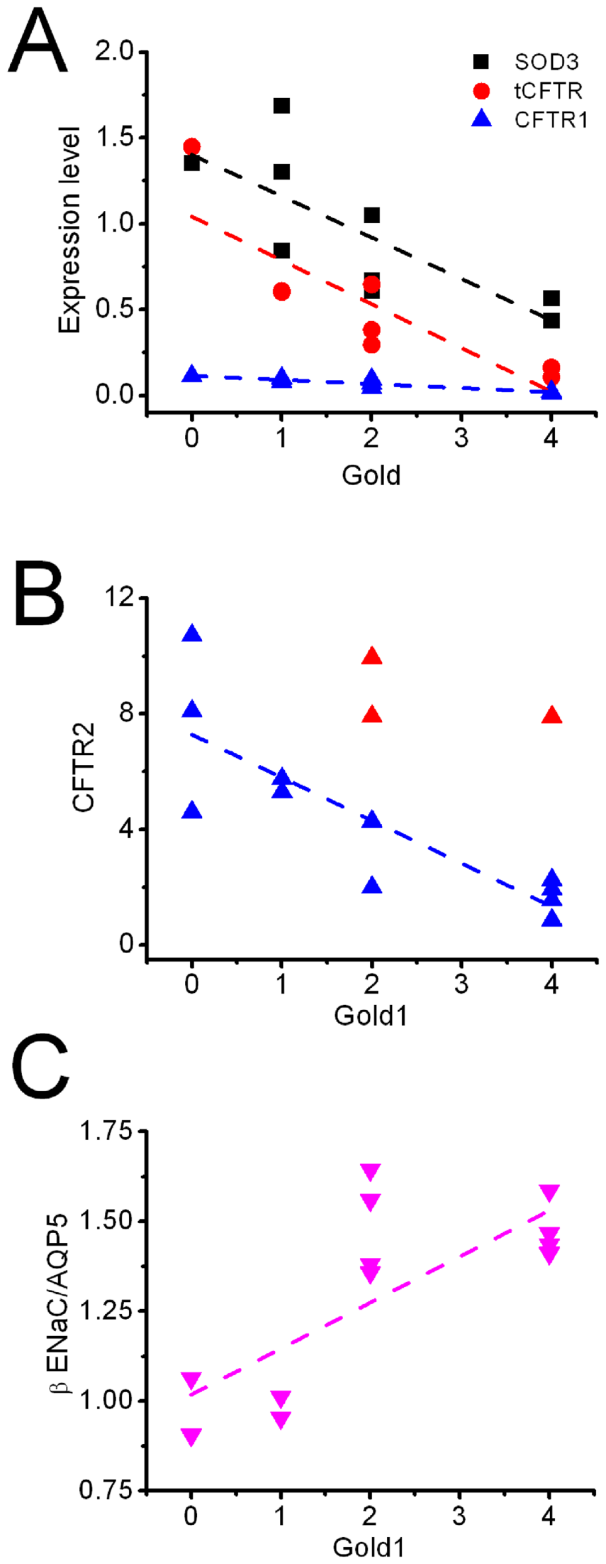
Expression of protein levels and COPD severity. **A**. SOD3, total CFTR (tCFTR), and CFTR in ATI cells (CFTR1) are associated with GOLD classification of COPD (see Methods for details). **B**. CFTR expressed in ATII cells correlates with GOLD1. **C**. β ENaC in ATI cells is positively associated with GOLD1.

Considering the significant correlations between examined proteins and spirometry tests, we reasoned that COPD severity also relates to the expression levels of these proteins. Analysis of protein densitometry showed that both total CFTR proteins and those in ATI cells correlated with GOLD stages (Figure 7A). SOD3 is a beneficiary biomarker - the more abundant the SOD3 proteins, the lesser the severity of COPD. CFTR in ATII cells also exhibited a linear regression with GOLD stage (Figure 7B). In sharp contrast to SOD3 and CFTR, the amount of β ENaC proteins was inversely associated with the severity of COPD stages (Figure 7C). The more the β ENaC proteins, the worse the lung function.

As is well-known, we found tight correlations between various clinical tests of lung function (data not shown). In addition, craniocaudal distribution of emphysema was apparently associated with lung function tests and SF12 evaluation (data not shown).

### Interactions of SOD3, pro-SPC, and channel proteins

Apically located ion/fluid transport proteins are exposed to ambient air and leukocytes. Reactive species regulate their functions via post-translational modifications, including oxidation, nitration, and nitrosylation [39], [40]. CFTR and ENaC interact with each other functionally and physically [41]. We postulated that in addition to the oxidative modifications, SOD3 affects their expression levels in COPD lungs. Our data showed that expression of SOD3 proteins is positively correlated with AQP5 (coefficient 0.644, P = 0.007), and CFTR (coefficient 0.804, P = 0.002). In stark contrast, an inverse association was found between SOD3 and β ENaC (coefficient −0.665, P = 0.005). Expression of the three ENaC subunits was associated with each other counterpart as anticipated (data not shown).

## Discussion

A pathological hallmark of COPD is obstructive mucus hypersecretion due to insufficient hydration of mucins. Over-expression and secretion of mucins draws more water from the watery sol layer, which is mainly governed by apically located ENaC, CFTR, and AQP5. This study aimed to investigate the expression levels of these proteins and their correlation with declined function in human emphysematous lungs. For the first time, our data demonstrate that ENaC translational levels are negatively associated with spirometric tests and diffusion capacity of carbon monoxide. Expression of AQP5 and CFTR, similar to SOD3, may benefit lung function and gas exchange in COPD patients. Besides, expression of ENaC, CFTR, and SOD3 is significantly associated with severity of COPD.

What is the relationship between ENaC translation and COPD? Accumulating evidence links ENaC to pulmonary diseases with imbalance between turnover and re-absorption of luminal fluid. It has been demonstrated that ENaC is a major determinant for the genesis of adult pulmonary edema and fluid resolution in mouse postnatal lungs (see reviews [36], [42]). Recently, emphysema and bronchitis were observed in transgenic mice overexpressing scnn1 genes [14], [18], [43]–[45]. The relationship between ENaC expression and lung function in COPD patients, however, was unclear. Our analyses for the first time demonstrated that the translation of ENaC in lung parenchyma is correlated with spirometry, gas exchange, and GOLD stages. Based on the correlation strength, an increment in the abundance of α and β ENaC proteins in ATI cells is strongly associated with FEV1 and DLCO, two critical manifestations of COPD. Combined with the preclinical studies in scnn1 transgenic mice, it is feasible to speculate that ENaC over expression may dehydrate luminal fluid both in the conducting airways and alveolar sacs. Mutated ENaC has been associated with gas diffusion in human subjects [46]. In addition, ENaC is expressed in human blood cells [47], [48]. Whether ENaC in erythrocytes regulates gas exchange and capacity is obscure. In toto, over-expression of ENaC proteins in COPD lungs could be a deleterious factor for lung function.

Expression of ENaC, AQP5, and CFTR is regulated by hormones, inflammation, and others individually. In addition, location of biopsy may vary detection of the expression level. Because the expression of these ion channel proteins is in a non-coordinate manner, it is explainable to see one of these proteins from some moderate and severe patients is expressed at normal level.

What are the implications of the association between the translational level of CFTR and lung function in COPD? CFTR was proposed as a genetic risk factor for COPD two decades ago [49]. The association between CFTR and COPD is emerging (see review [34]). This study demonstrates that CFTR proteins expressed in ATI cells are positively associated with FEV1 and FVC in COPD patients. These observations are supported by recent publications [35], [50]. In addition to functioning as a channel for anion/fluid permeation, CFTR regulates ceramide signaling and lipid rafts in emphysematous lungs [35], [51]. Reduction in CFTR expression in COPD lungs could be associated with up-regulation of ceramide signaling, which might stimulate the release of neutrophilic elastase and myeloperoxidase, which in turn would lead to alveolar enlargement. A beneficial role of CFTR is evidenced by the association with lung function in parallel with SOD3, a well-known preventive molecule against COPD. CFTR has been reported to play an important role in alveolar fluid clearance in non-cystic fibrosis lungs. We thus believe that impaired CFTR in COPD lungs may contribute to dehydration and occluded airways.

AQP5, as a rate-limiting barrier for transepithelial water flow, is located in airway epithelial and ATI cells [37], [52]. AQP5 transcripts were found in the bronchial tissues and were positively correlated with FEV1 in COPD patients [38]. Whether the expression of CFTR proteins is associated with spirometry and other clinical measures is unknown. This study found that abundance of AQP5 in COPD lungs is significantly correlated with both spirometry tests and gas diffusion. Considering that AQP5 is expressed in ATI cells and contributes up to 93% of the surface area of alveoli, reduction in AQP5 expression may result in loss of ATI cells and dehydration of air sacs. Both surface area and fluid homeostasis have been confirmed to alter lung function and gas diffusion.

In conclusion, our observations for the first time confirm that ENaC, CFTR, and AQP5 are closely correlated with clinical lung function, indicating their novel roles as potential biomarkers in the pathogenesis of human COPD [53]. Similar to SOD3, CFTR and AQP5 may be beneficial molecules against COPD. Up-regulation of CFTR and AQP5 expression, therefore, could be therapeutic interventions for COPD. Correction of excessive expression of ENaC proteins in COPD may be a potent therapy as well.
